# Supplementary material for: Study protocol for the BUSCopan in LABor (BUSCLAB) study: A randomized placebo-controlled trial investigating the effect of butylscopolamine bromide to prevent prolonged labor
Source: PLoS One. 2022 Nov 3;17(11):e0276613. doi: 10.1371/journal.pone.0276613 (PMC9632812; doi:10.1371/journal.pone.0276613)
Supplement: S2 File — (PDF) [file pone.0276613.s002.pdf]

A new measure to prevent slow labor progress

To women who are going to give birth to their first child at Rikshospitalet
Would you like to take part in a study to test if the drug Buscopan can reduce labor duration?

Why are we conducting this study?
The staff at the maternity ward want to help you in the best possible way. Among other things, we are concerned that labor should not last too long. Therefore, we want to try a new and easy way to shorten labor if it turns out to be progressing slowly. Research shows that labor can be shorter with Buscopan, a drug that is commonly used to treat various types of abdominal pain.

Who can participate?
You can participate in the study if your baby is lying with its head down and labor starts spontaneously after pregnancy week 37. If your cervix opens more slowly than 1 cm per hour after 3 cm opening, you can participate in randomization (lottery) deciding whether you will be given Buscopan (active ingredient) or saline solution (placebo). Randomization is the best way to determine if a drug is effective.

What does the study entail?
Buscopan / placebo will be given by a small plastic tube that is inserted into a vein in your hand by a thin needle. Such an intravenous access will be required during most deliveries anyway, for example in connection with epidural anesthesia or intravenous fluid.
The first half hour after you receive the medication you will be connected to a monitor (CTG) that records your and your baby's pulse. Buscopan can also relief pain, so you will be asked to rate the pain before and after the drug / placebo is given. We will record information from your medical record about the birth and information of past illnesses that are of significance for care during labor and birth. You will be sent a birth experience questionnaire one month after birth, which is voluntary to respond to.

Who will get the treatment?
Half of the participants receive the drug, the other half receive saline solution. Neither patient, midwife nor doctor knows who gets what. If labor progresses so slowly that measures according to the department's guidelines should be taken (less than 1 cm in 4 hours), these will be implemented according to normal routine, whether you participate in the study or not. If this has not happened yet, your waters will be artificially broken (Artificial rupture of membranes, ARM) and you will be given an intravenous drip to stimulate contractions (oxytocin infusion). You are thus receiving the same treatment regardless of participation in the study.

Possible advantages, disadvantages and side effects
Buscopan seems to be relaxing the cervix and the opening may be faster. This drug acts in a different way from oxytocin (iv drip), which gives you stronger contractions. If the opening goes faster, there is less chance that you will need oxytocin later in childbirth. Buscopan is given as a single dose within one minute, while oxytocin is usually given continuously over several hours. When receiving oxytocin, you must always be connected to a CTG monitor. Possible side effects of Buscopan include dry mouth, rapid heart rate, short period of dizziness and lower blood pressure. The drug has been tested on 2,600 women in labor in 19 studies without any serious side effects, neither for mother nor infants. Regional Center for Drug Information (RELIS) state that Buscopan passes the placenta only to a small extent, and that there is little reason to believe that Buscopan given to the mother affects the baby.

What happens to the information about you?
All information about you will be processed unidentifiable, with no name or birth number or other directly recognizable information. Only personnel responsible for the study have access to the information. It will not be possible to identify you in the results of the study when they are published.

Voluntary participation
Participation in the study is voluntary. You can withdraw from the study at any time, without giving any reason, and without any consequences for your further treatment. If you wish to participate you can bring the signed form to the maternity ward. If you are uncertain, you can wait to decide (and sign) until you reach the maternity ward and know more about how fast your birth is progressing.

On this page you will find: further information about the study in Chapter A (below) - *a detailed explanation of what the study entails***. Further information on biobank, privacy, finance and insurance in Chapter B -** *Privacy, biobank, finance and insurance.*
Statement of consent follows Chapter B *Signed by the person who consents to participate in the study. The person, who has informed about the study, can confirm that the information has been provided.*

Chapter A - detailed explanation of what the study entails First-time mothers who will give birth at Oslo University Hospital Rikshospitalet are invited to participate in the study. The birth must start spontaneously, without the use of oxytocin and the duration of pregnancy must be over 37 weeks. Women with a baby in breech position or twin pregnancies cannot participate. Women showing signs of slow progress in childbirth, i.e. less than one cm opening per hour after the cervix is open 3 cm, are randomized to Buscopan or placebo. Only one dose (1 ml) is given. In addition, all participants receive the standard treatment of the department regardless of whether they receive active medication or placebo.

Previous research has mostly been done in other parts of the world and in these Buscopan was been given to all women in labor, not just those with slow progress. There are 19 randomized studies, involving a total of 2,600 women worldwide who have been given Buscopan or similar drugs in childbirth to investigate whether the lenght of labor is shortened. None of these studies have reported serious adverse effects for mother or child. Overall, the studies indicate that labor is shorter in the women who received Buscopan (Rohwer AC and co-workers, Cochrane 2013). However, no one has investigated whether Buscopan is effective in helping women whose birth is slower than expected. It is estimated that we will need about 125 patients in each group (drug / placebo) to be able to show differences with and without Buscopan in this study. All participants will be followed up with a birth experience questionnaire four weeks after birth. The study participants are covered by insurance in accordance with ” Lov om produktansvar i Legemiddelforsikringen.”

Chapter B - Privacy, bio bank, finance and insurance. Privacy and right of access and storage of material Financing and insurance. The Norwegian Medicines Agency and control authorities can be provided with study information and given access to relevant parts of your journal. The purpose is to check that the study information matches the corresponding information in your journal. Everyone who has access to information about you has a duty of confidentiality. If you agree to participate in the study, you have the right to have access to the information that is registered about you. You also have the right to correct any errors in the information we have recorded. If you withdraw from the study, no further information or material will be collected. Information already collected from you will not be deleted. The study is funded by Oslo University Hospital and Health South-East. You are insured under “Lov om produktansvar i Legemiddelforsikringen”.

Information on the outcome of the study. Approval. Contact Information. All participants have the right to receive information on the results of the study. The project is approved by the Regional Committee for Medical and Health Research Ethics (case number 2018/2380). Under the new Personal Data Act, Oslo university hospital and project manager, chief medical officer Trond Michelsen, has an independent responsibility for ensuring that the processing of your information has a legal basis. This project has a legal basis in Articles 6a and 9a of the EU Privacy Policy. You have the right to complain about the processing of your information to “Datatilsynet”. Oslo University Hospital is responsible for the study. You can contact the institution's privacy representative if you have questions about the processing of your personal data in the project, personvern@ous-hf.no, tel: 915 02 770.

If you have questions about the study, contact midwife and Phd student Lise Gaudernack (lisgau @ ous-hf.no, phone 230 72 656/230 72 640) or project manager and chief medical officer Trond Michelsen (trmi1@ous-hf.no).

Consent to participate in the study: I want to participate in the study (participant)
Date: Sign:
-- -- ------------
I confirm having provided information about the study (midwife)
Date: Sign:
--
