# Supplementary material for: Study protocol for the BUSCopan in LABor (BUSCLAB) study: A randomized placebo-controlled trial investigating the effect of butylscopolamine bromide to prevent prolonged labor
Source: PLoS One. 2022 Nov 3;17(11):e0276613. doi: 10.1371/journal.pone.0276613 (PMC9632812; doi:10.1371/journal.pone.0276613)
Supplement: S4 File — (PDF) [file pone.0276613.s004.pdf]

Dette er en e-post sendt automatisk fra Damnett og kan ikke besvares (EW 5B.1).

Prosjekt 2020/FO283405 Buscopan ved langsom fødsel - en RCT har fått tildelt midler ved årets søknadsbehandling. I alle forskningsprosjekter skal det inngås kontrakt mellom søkerorganisasjon, forskningsinstitusjon og forsker/stipendiat som omhandler rettigheter, plikter og økonomiske disponeringer. Når prosjektkontrakt er signert skal denne lastes opp i Damnett. Oppgaven Bekrefte prosjektkontrakt ligger nå klar.

Oppgaven utfører du ved å logge deg inn på https://www.damnett.no. Husk å lese brukerveiledningen. Du finner en lenke til denne nederst på vår innloggingsside eller inne i Damnett under Hjelp-funksjonen.

Det er ikke laget en standardkontrakt så hver organisasjon må selv legge inn de krav som gjelder i en kontrakt, både krav fra Stiftelsen Dam og organisasjonenes egne betingelser.

Det genereres også en oppgave Bekrefte prosjektkontrakt for bevilgninger år 2 og 3. Det er for de prosjektene som inngår kontrakt for ett år av gangen eller fornyer kontrakten hvert år. Denne er ikke obligatorisk hvis det er inngått kontrakt første året for hele prosjektperioden.

Ved andre spørsmål kan Stiftelsen Dam kontaktes.

Vennlig hilsen

Stiftelsen Dam
