# Supplementary material for: Study protocol for the BUSCopan in LABor (BUSCLAB) study: A randomized placebo-controlled trial investigating the effect of butylscopolamine bromide to prevent prolonged labor
Source: PLoS One. 2022 Nov 3;17(11):e0276613. doi: 10.1371/journal.pone.0276613 (PMC9632812; doi:10.1371/journal.pone.0276613)
Supplement: S5 File — (PDF) [file pone.0276613.s005.pdf]

Trond Michelsen
Oslo universitetssykehus
trmi1@ous-hf.no

Trondheim, 10. mai 2019

Tildeling av forskningsmidler fra DKNVS

Det Kongelige Norske Videnskabers Selskab (DKNVS) har i styremøte 29. april 2019 besluttet å støtte ditt prosjekt «BUSCLAB - A double blind randomized placebo-controlled trial investigating the effect of intravenous Butylscopolamine bromide to prevent slow progress in labor» med **kr 30 000** gjennom DKNVS Stiftelses forskningsfond.

Det kom inn totalt 64 søknader til årets utlysning fra DKNVS Stiftelses forskningsfond. Ifølge utlysningsteksten skal midlene fra dette fondet fordeles på inntil fem mottakere, men ut fra en samlet vurdering, basert på antall søknader, kvaliteten på disse og nivået på de omsøkte beløpene, vedtok styret å fordele beløpet på ni mottakere. Støttebeløp: fra kr 10 000 til kr 40 000.

Vennligst send oss kontonummer og prosjektnummer for utbetaling av midlene.

Rapportering og kreditering:

- Det Kongelige Norske Videnskabers Selskab (DKNVS) skal bli kreditert i alle publiserte arbeider fra det støttede prosjektet.
- Når prosjektet er avsluttet, ber vi om å få tilsendt en kort rapport fra arbeidet som vil bli benyttet i årboka. Formkrav til rapporten fås ved henvendelse til DKNVS' administrasjon.
- Vi mottar også gjerne den fullstendige prosjektrapporten til oppbevaring i våre arkiver.
- Du kan bli forespurt om å gi muntlige presentasjoner fra prosjektet i DKNVS' forskningsformidlingsarenaer.
- Dersom prosjektet endrer seg i nevneverdig grad eller ikke gjennomføres, kan DKNVS kreve tilbakebetaling av de tildelte midlene.

Se oversikt over støttede prosjekt på www.dknvs.no/sok-stotte/tildeling-2019/.

Vi ønsker lykke til med prosjektet!

Vennlig hilsen
Det Kongelige Norske Videnskabers Selskab

Kristian Overskaug

Kristian Overskaug
Generalsekretær
