# Supplementary material for: Study protocol for the BUSCopan in LABor (BUSCLAB) study: A randomized placebo-controlled trial investigating the effect of butylscopolamine bromide to prevent prolonged labor
Source: PLoS One. 2022 Nov 3;17(11):e0276613. doi: 10.1371/journal.pone.0276613 (PMC9632812; doi:10.1371/journal.pone.0276613)
Supplement: Attachment — Submitted filename: Response to Reviewers.docx [file pone.0276613.s006.docx]

To: Staff Editor Jianhong Zhou

PLOS ONE

Resubmission date: August 3rd, 2022

Original Submission date: December 4th 2021

Dear Dr. Jianhong Zhou,

Thank you for the thorough review from Editor and reviewers considering our submitted manuscript entitled: “Study protocol for the BUSCopan in LABor (BUSCLAB) study: A randomized placebo-controlled trial investigating the effect of butylscopolamine bromide to prevent prolonged labor”.
We have revised the manuscript according to the valuable suggestions made and we believe the manuscript has improved considerably.
Our responses to the comments are listed below point-by- point, with a description of the changes made to the manuscript.

The word count is now 4072 words.

Best regards,

Dr. Ingvil Krarup Sørbye, corresponding author

**Response to Reviewers**

| Editor/Reviewer’s Comments: | Authors’ Comments: |
| --- | --- |
| **Journal Requirements:**  When submitting your revision, we need you to address these additional requirements.  1. Please ensure that your manuscript meets PLOS ONE's style requirements, including those for file naming. | Thank you for your valuable and useful comments.  We have now checked that the revised manuscript adheres to the requirements of PLOS ONE’s style. |
| 2. Please include a copy of Table 1 which you refer to in your text on page 13. | Sorry about this mistake: we meant to refer to Figure 1 in the text on page 13. |
| 3. Please include captions for your Supporting Information files at the end of your manuscript, and update any in-text citations to match accordingly. Please see our Supporting Information guidelines for more information: <http://journals.plos.org/plosone/s/supporting-information>. | We have included captions for Supporting Information files in the manuscript according to the guidelines (S1-S5 Files) (Page 26). |
| **Review Comments to the Author** |  |
| **Reviewer #1:** |  |
| This study focus on prolonged labour and the use of buscopan.  The study aims to evaluate the duration of labour when using buscopan for prolonged labour comped to placebo.  General:  Why is it important to reduce duration of labour? Why is this the most important outcome, the primary outcome of your study?  Does the women prefer a 60 minutes reduction? Does the clinicians? | In this study we want to assess any effect of butylscopolamine on labor progress in a cohort of women with slow progress of labor, defined as the crossing of the partograph alert line. The choice of the primary outcome of duration of labor was therefore relevant, as long labors are associated with a range of adverse outcomes including a negative birth experience. We agree that other outcomes, such as operative delivery, are highly relevant and could have been chosen as main outcomes; however, this would have implicated the inclusion of a much larger cohort due to statistical power issues.  The reviewer rightly points out that a reduction of 60 minutes duration of labor is an arbitrary cut-off, and the cut-off can certainly be discussed, but has been used in recent systematic reviews (Riemma G et al. Efficacy of hyoscine butyl-bromide in shortening the active phase of labor: Systematic review and meta-analysis of randomized trials. Eur J Obstet Gynecol Reprod Biol. 2020 Sep;252:218-224. doi: 10.1016/j.ejogrb.2020.06.042. Epub 2020 Jun 23. PMID: 32629224). In this trial we include women who show early signs of long labor and thus are at risk of adverse outcomes. If our trial shows that butylscopolamine bromide shortens labor in this population, larger studies may be warranted to look at more robust clinical maternal and fetal endpoints. |
| Why is the study updated as completed at clinicaltrials.gov? Have you already terminated the inclusion? | We submitted our study December 4^th^ 2021 to PLOS ONE, thus seven months ago. At that time we were still including participants. After several reminders, we received a decision from PLoS One on July 11th 2022. We have since recently finalized inclusion of the planned number of participants. |
| Consider using SPIRIT checklist when presenting a trial protocol – the chronology of paper could be improved by the use of spirit.  You repeat yourself several times during the paper. Please do only report eg. the objective and the outcome measures once.  Try to be more concise – current the word count is very high. | Thank you for this suggestion. We have made edits according to the chronology of the SPIRIT checklist (S1 File).  We have removed unnecessary repetitions and hence shortened the revised manuscript.  We have now reduced the word count from 4565 words to 4072 words. |
| Introduction:  A definition of prolonged labour is lacking, also definition of active phase should be included in the introduction.  “Prolonged labour is common”, could you quantify? | Thank you for noticing this. We have now added a definition of prolonged labor and of active phase of labor (Abstract, page 4, lines 48-49 and Introduction, page 6, line 76). We have also added a sentence regarding how common prolonged labor is among nulliparous women (Abstract, page, line 49 and Introduction, page 6, line 74). |
| Oxytocin- suggest that you focus on the primary adverse effect of oxytocin: uterine hyperstimaultion (tacycysystole) which may lead to compromised oxygen supply for the baby, fetal asphyxia and the need for immediate delivery. Other adverse effects could also be mentioned (postpartum hemorrhage, water intoxication, uterine rupture). The effect on breastfeeding remains as far as I am aware still unknown.  Instead of criticizing oxytocin you might consider focusing on the synergistic effect of two different drugs? | Thank you for this suggestion. We have changed the Introduction to focus more on the primary adverse effects of oxytocin. We have also modified the statement regarding the effect of oxytocin on breastfeeding (Introduction, page 6, lines 84-87 and lines 87-89).  We have added a sentence regarding a possible beneficial synergistic effect of the two different drugs in the Discussion, page 19, lines 381-382. |
| Prolonged labour – should also lead to the considerations as to whether there are other explanations as to whether labour do not progress – e.g. fetal/pelvic disproportion | Thank you for this comment, which we agree with. Cephalopelvic disproportion is a cause of prolonged labor, as is ineffective uterine propulsive forces. The present study assesses time from IMP to delivery as the primary outcome. Hence, the cause of dystocia will not be evaluated as part of the primary outcome. However, we do not expect that women will cephalopelvic disproportion will have effect of butylscopolamine. We have estimated that 8% of participants will deliver by emergency cesarean section (section on Power Analysis). Likely, most participants with cephalopelvic disproportion will delivery by cesarean section and will be censored. However, we plan to study causes of prolonged labor and the effect of butylscopolamine in exploratory analyses later in the project. |
| Study overview:  Your hypothesis and objective should be merged into one sentence. And comnsider only to report the primary objective of the trial (like a PICO presented as a sentence)  You should report primary outcome and secondary outcomes, not ‘objectives’ | Thank you for this suggestion. We have rephrased as suggested and now report outcomes and not objectives (Introduction, page 8, lines 139-143) and Methods, pages 11-12, lines 212-226). |
| Patient enrollment and eligibility criteria:  Please define the active phase for your study earlier in the text or at least mention in the introduction the variety of different definition. Do you only consider cervical dilatation to be relevant, not contractions, amniotomy, cervical length? | Thank you for this suggestion. We now define the active phase earlier in the Manuscript (see comment above) Introduction, page 6, line 81). |
| Please consider moving ref 26 and 27 to the introduction | We have moved the two references to the Introduction (Introduction, page 6, lines 78-80). |
| No need to both text the inclusion criteria and exclusion criteria and also put the in a box. Choose one of the two options. | Thank you for this suggestion. We have removed the inclusion and exclusion criteria from the main text and kept them in the box. |
| Sample size calculation:  Consider a brief presentation of the Dencker trial in order to help the reader, they randomised, but to which intervention?  Please consider to describe your samplesize calculation with less word, more consiese. | Thank you for this suggestion. We have added information about the Dencker trial (Methods, page 12, lines 228-239). We have rephrased and condensed the paragraph regarding the sample size calculation (Methods, page 12, lines 228-242). |
| Is the trial GCP monitored? If yes please report that, if not please argue why not | The BUSCLAB trial will be GCP monitored by the independent Clinical Trial Unit located at Oslo University Hospital. We have added this information in Methods, pages 16-17, lines 328-333. |
| **Reviewer #2:** |  |
| The manuscript needs a language editor. There are so many typographical errors. | Thank you for noticing this. We have had a professional language check performed. |
| ABSTRACT  The objectives of the study is not well defined.  The authors stated: Women are included in the first stage of labor if they cross the partograph alert line with a cervical dilation between 3–9 cm. Does it mean that authors recruit patients at 9cm cervical dilatation? If yes, would that not be too late? | We have rephrased the description of the objective of the study (Introduction, page 9, lines 146-149). We chose to include women if they crossed the alert line of the partograph and with a cervical dilation between 3-9 cm, as this defines the active first stage of labor in our facility. It is plausible that an effect of butylscopolamine could be relevant also for women towards the end of the first stage. Previous studies and metaanalyses have reported shorter first stage of labor in participants who received butylscopolamine. However, previous studies did not address which part of the first stage of labor that was shortened. Thus, we chose to include women with up to 9 cm dilatation in our study. We argue that another cut-off earlier in the first stage of labor would be arbitrary. Still, we expect that few participants will be included as late as at 9 cm dilation. |
| INTRODUCTION  The authors stated: Another class of drugs, antispasmodics, has been used to prevent poor progress of labor in both developing and developed countries. The authors should replace developed to 'high-income' and replace developing to 'low and middle-income' | Thank you for this suggestion, which has been implemented (Introduction, page 7, lines 96-97). |
| **Reviewer #3: Comments to the Author** |  |
| I read with great interest the enclosed Manuscript which falls within the aim of Plos One.  In my honest opinion, the topic is interesting enough to attract the readers’ attention. Methodology is accurate.  However, it is necessary to cite and discuss previous trials and meta-analyses regarding the aim of trial, since there are several trials on the issue. See and cite this updated meta-analysis which summarizes the available evidence from RCTs, PMID: 32629224. | Thank you for your comment.  We have included the most recent meta-analysis in the Introduction (page 8, lines 128-134) and in the Discussion (page 19, lines 379-380 and lines 383-384), as suggested. |
